# An exploratory study of patient hospitalization patterns and behavioral risk factors using mobile phone location data

**DOI:** 10.1371/journal.pdig.0001512

**Published:** 2026-07-23

**Authors:** Takeshi Uchikura, Kosuke Takata, Masayuki Yoshikawa, Hokuto Morohoshi, Shunichi Ishii, Tomiko Sunaga, Kenji Momo

**Affiliations:** 1 Department of Hospital Pharmaceutics, School of Pharmacy, Showa Medical University, Tokyo, Japan; 2 Department of Hygiene, Public Health and Preventive Medicine, School of Medicine, Showa Medical University, Tokyo, Japan; 3 Division of Applied Pharmaceutical Education and Research, Hoshi University, Tokyo, Japan; University of Bristol, UNITED KINGDOM OF GREAT BRITAIN AND NORTHERN IRELAND

## Abstract

This exploratory observational study examined associations between behavioral patterns and hospitalization using geographic analysis of human mobility data. Anonymized GPS-based location data were obtained from users of a mobile location data platform in the Tokyo South Medical Region (Shinagawa and Ota wards) between April 1, 2022, and February 28, 2023. Behavioral patterns, including eating-out frequency and healthcare facility visits, were inferred from movement data. Hospitalization was defined as a hospital stay of at least two consecutive days, identified based on location trajectories. The primary endpoint was the association between eating-out behavior and hospitalization during the observation period. Logistic regression models were used to estimate odds ratios (ORs) and 95% confidence intervals (CIs). Venn diagrams were used to illustrate overlapping behavioral characteristics between groups, complementing regression-based analyses by visualizing behaviors that are difficult to capture through average regression effects and by supporting hypothesis generation. In total, 647 participants were included, of whom 580 were classified as outpatients, whereas 67 experienced hospitalization during the study period. Higher odds of hospitalization were observed only among participants who ate out more than eight times per month, compared with those who ate out less frequently (OR = 1.89, 95% CI: 1.05–3.43). No clear association was observed for the other eating-out frequency categories, with confidence intervals that included the null value. Venn diagram analyses suggested that individuals with both frequent outpatient visits and frequent eating-out behavior were more prevalent in the hospitalized group. These findings demonstrate the potential utility of mobile phone-derived mobility data for identifying behavioral patterns associated with hospitalization. However, given the observational design, reliance on indirect behavioral proxies, and missing demographic information (including age and sex), the results should be interpreted with caution. Further studies integrating detailed clinical and demographic data are needed to clarify causal relationships and evaluate the applicability of mobility-based behavioral indicators in healthcare settings.

## Author summary

Understanding the correlation of everyday behaviors to health outcomes is important for preventing avoidable hospitalizations. Herein, we examined whether patterns of daily movement captured through anonymized mobile phone location data could help identify behavioral pattern associated with hospitalization. We analyzed geographic mobility data from individuals living in the Tokyo South Medical Region between April 2022 and February 2023. By estimating the frequency of visits to restaurants and healthcare facilities, we compared behavioral patterns between those who were hospitalized and those who were not. We found that only individuals who ate out frequently (more than eight times per month) had a higher likelihood of hospitalization compared with those who ate out less often, even after accounting for other behavioral patterns. Hospitalized participants were more likely to both visit healthcare facilities regularly and ate out frequently. While this observational study does not establish cause-and-effect relationships, it highlights the potential value of mobile phone-derived mobility data for understanding real-world behavioral factors linked to health outcomes. Such approaches could complement traditional clinical information and help support future efforts to identify individuals who may benefit from early lifestyle guidance or preventive interventions.

## Introduction

Healthy food intake, adequate life style including exercise, and good sleep are undeniable health-related factors. In healthcare research, several studies have reported lower risk for death and dementia in dog owners [1,2]. A consistent finding across these reports suggests that dog owners benefit not only from the mental stability based on companionship with their dogs but also from the physical activity of walking their dogs [3]. Conversely, negative health outcomes such as increased mortality [4], obesity [5–7], cardiovascular diseases [8], diabetes mellitus [9] have been associated with habitually eating out. During the COVID-19 pandemic, lockdown-imposed restrictions further contributed to unhealthy behavior [10]. These patterns suggest that healthy habits can be predicted with considerable accuracy from people’s moving patterns. In other words, monitoring people’s movements can serve as a surrogate marker for their health-related habits. During the COVID-19 pandemic in Japan, population behaviors were strongly influenced by public health measures. Repeated states of emergency and government requests to reduce mobility and shorten business hours remained in place until early 2022. The study period (April 2022–February 2023) therefore represents a transitional phase in which most formal restrictions had been lifted and daily life was gradually returning toward prepandemic conditions. Nevertheless, individual behavioral patterns may still have been affected by residual pandemic-related influences. Examining behavioral patterns during this transitional period is therefore meaningful for understanding the correlation of population-level mobility to health outcomes.

In the decade, geographic analysis of human movement has advanced alongside the development of mobile phone equipping with global positioning system (GPS) technology. Concept such as predicting traffic congestion [11], efficiently operating public transportation systems [12], formulate evacuation plans during disasters [13], and assessing the effects of air pollution [14] have become increasingly integrated in society.

In health-care fields, beyond the aforementioned studies, digital therapies using smartphone application are being introduced on an individual level. However, behavioral analysis targeting population as “big data”, without restricting to specific disease groups or demographics, has not been sufficiently conducted. Therefore, we conducted an exploratory survey using geographic analysis of human movement to investigate behavioral patterns associated with hospitalization.

## Methods

### Ethics statement

The data were obtained from Blogwatcher, Inc. on October 6, 2023, and consisted of anonymized processed information in compliance with Japan’s Act on the Protection of Personal Information. Accordingly, individual informed consent was not required for data provision or use. Under the Ethical Guidelines for Clinical Research in Japan [15], studies using anonymized processed information do not require review by an institutional ethical review committee. All methods were performed in accordance with relevant guidelines and regulations.

### Study definition

Geographic information was collected via GPS, pinpointing participants locations with ±1–5 m accuracy through smartphone tracking every 5–15 min via installed applications running in the background. Locations where participants stayed were overlaid onto maps to visualize their positions. The study covered by the Tokyo South Medical Region (Shinagawa and Ota wards) (S1 Fig) from April 1, 2022 to the end of February 2023.

A “temporary visit” was defined as staying at one location for >15 min; however, the precise duration of these stays was not included in the dataset. Admission history was identified as a hospital stay of at least two consecutive days. “Employment” was defined as a location other than the assumed residence with the highest geographic information data during daylight hours. Various location types were identified from map information, including eating establishments, gambling venues, clinics/hospitals, supermarkets/shops, pharmacies, and health-related administrative facilities (health centers, etc.). (S1 Table)

### Study design and endpoint

This exploratory observational study used anonymized geographic information to investigate the behavioral patterns associated with hospitalization.

The primary endpoint was the association between habitual eating-out behavior and hospitalization during the observation period, because eating-out patterns were considered a mobility-derived behavioral marker potentially related to cardiovascular health. The secondary endpoint aimed to identify the differences in movement behaviors (staying at home vs. visiting gambling establishments) between individuals with and without a history readmission. For individuals in the admission group, eating-out frequency was calculated using data from the entire observation period and was not limited to the period before hospitalization. This approach was adopted because, given the small number of cases, including hospitalization dates could have increased the risk of re-identification through linkage with other data sources. Therefore, hospitalization dates were excluded during anonymization in accordance with Japan’s Act on the Protection of Personal Information. Accordingly, eating-out frequency was interpreted as a behavioral pattern observed during the study period rather than as a temporally defined pre-hospitalization exposure.

### Data source

The mobile phone location information used in this study was obtained from Blogwatcher, Inc. as anonymized processed information. Blogwatcher, Inc. has 25 million users (devices) with at least one use or activity per month, representing approximately 12% of all smartphone devices in Japan [16]. Location data was obtained only from users who granted permission through more than 140 different smartphone applications, such as transit information, restaurants guide, and electronics stores apps. All data was collected after obtaining the user permission for GPS information tracking. Although smartphone ownership exceeds 97% in Japan, the dataset does not include individuals without mobile phones (approximately 3%). In addition, individuals with little or no mobility, such as homebound or bedridden patients, estimated to number approximately 310 000 nationwide, were not captured because they do not generate movement records. While individual-level socioeconomic information was not available in the dataset, smartphone ownership has become widespread in recent years, including among individuals receiving public assistance.

### Case identification (Outpatient group and admission group)

**Fig 1:**
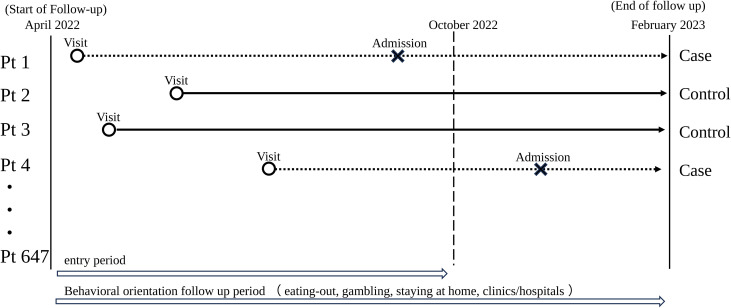
Diagram illustrating the identification and follow-up of cases Case: admission group, Control: outpatient group.

This study focused on cases receiving follow-up care. The Showa Medical University Hospital and cardiovascular-specific clinics or hospitals registered by the Japanese Society of Cardiology [17] were identified in the Shinagawa and Ota regions of Tokyo. The “outpatient group (control)” was defined as individuals meeting the following criteria: (1) visited or stayed to Showa Medical University hospital at least 1 d between April 1, 2022, and October 31, 2022, and (2) visited Showa Medical University Hospital and cardiovascular-specific clinics/hospitals for at least 1 d. The “admission group (case)” met criteria (1) and (2), along with (3): a hospital stay of at least 2 d within the Tokyo South Medical Region (Fig 1).

### Data analysis

In this study, to account for varying follow-up durations across participants, person-months were calculated as the sum of the follow-up time of each participant in months, measured from the start of observation to censoring at hospitalization, loss to follow-up, or the end of the study. Person-months were used as the denominator when reporting event rates. Logistic regression models analyzed behavioral factors related to hospitalization and calculated odds ratios with 95% confidence interval (ORs [95% CI]). Explanatory variables included eating out frequency, time spent at home, monthly clinics/hospital visits, visits to gambling establishments, and employment status. The threshold for eating out was determined based on data from the National Health and Nutrition Survey in Japan, 2019 [18]. Data analysis was performed using JMP 17 (SAS Institute Inc., Cary, NC, USA). Venn diagrams were additionally used to illustrate overlapping behavioral characteristics between groups, visualizing combinations of behaviors that are difficult to capture using average regression effects and supporting hypothesis generation. These included eating-out patterns, time spent at home, visits to clinics or hospitals, and visits to gambling establishments, thereby complementing the regression-based analyses.

## Results

### Participant characteristics

We identified 647 participants during the study period from mobile phone location information. Of these, 580 participants met the criteria for the “outpatient group”, whereas 67 participants met the criteria for the “admission group”. Age information was available for 45.3% of the outpatient group and 47.8% of the admission group (Table 1). In the outpatient group, participants were represented across all age categories among those with available age data, whereas no participants aged 20–29 years were identified in the admission group. For employment status, relatively few participants in either group were classified as employed. The behavioral Orientation results for both groups are shown in S2 Table. The small size of the admission group along with the substantial proportion of missing demographic data, means it is not possible to compare demographic characteristics of the outpatient and admission groups.

**Table 1:** Patient background.

	outpatient group	admission group
Number	580	67
Male/female/ not available	136/127/317	17/15/35
Age		
20-29	9	0
30-39	24	3
40-49	47	8
50-59	76	7
60-69	48	7
70-	61	7
not available	315	35
Employment Yes/No	269/311	31/36

*The relevant information was not available

### Relevance of behavioral orientation (Primary/Secondary endpoint)

**Fig 2:**
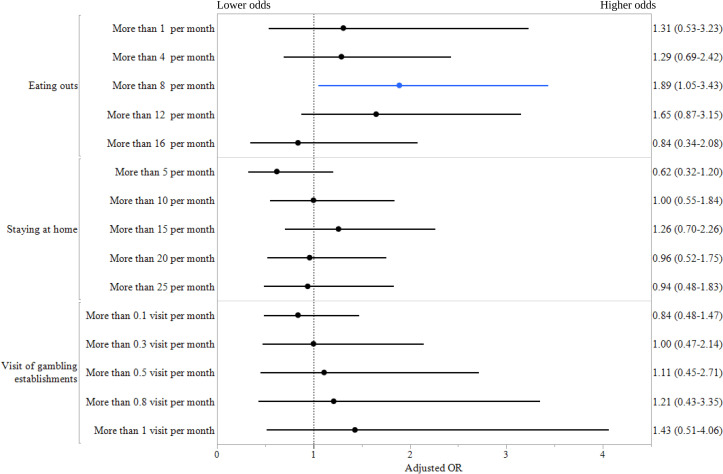
Behavioral factors associated with patient hospitalization.

In our exploratory analysis, higher odds of hospitalization were observed only among participants who ate out more than eight times per month, compared with the low-frequency group (OR = 1.89, 95% CI: 1.05–3.43) (Fig 2, S3–S5 Tables). For the secondary endpoint of visits to gambling establishments, higher visit frequency was associated with numerically higher odds; however, the confidence intervals were wide and included the null value.

### Characteristics of behavioral orientation (Venn-diagrams)

**Fig 3:**
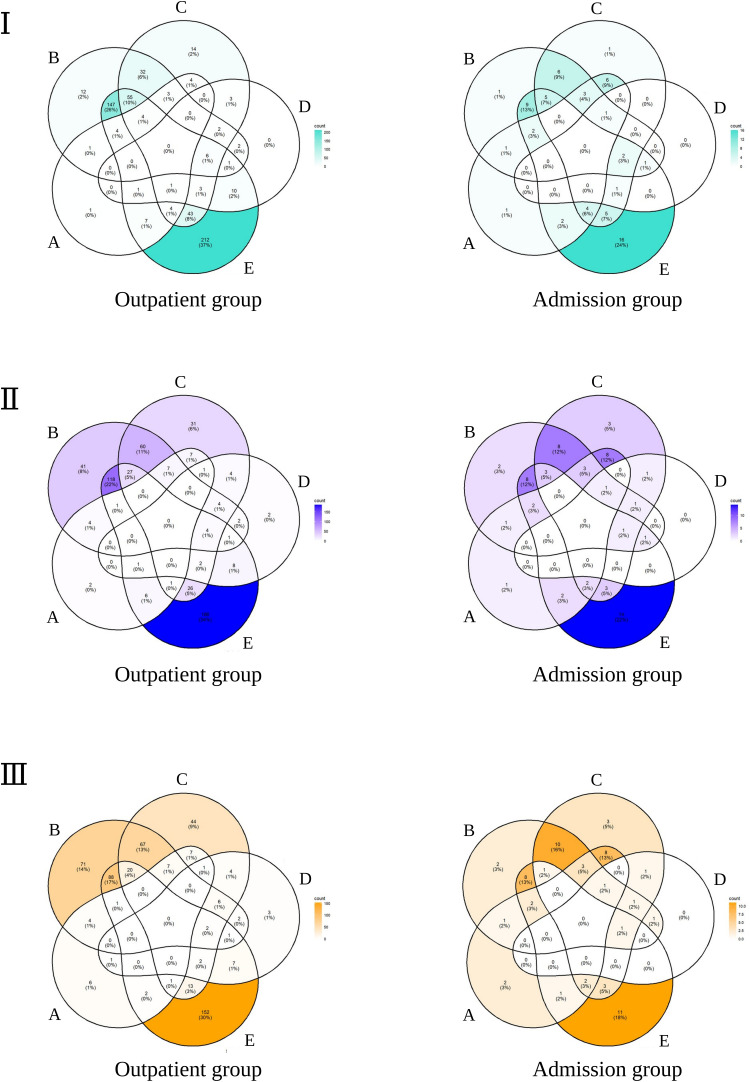
Venn Diagram on Cases Characteristics of Behavior. A: Monthly clinics/hospitals visit, B: Employment, C: More than eight eating outs outings per month, D: More than one visit per month to gambling establishments, E: Staying home (Ⅰ: More than 5 per month, Ⅱ: More than 10 per month, Ⅲ: More than 15 per month).

To understand the overlapping behavioral characteristics of participants eating out more than eight times per month, we created three Venn diagrams based on the frequency of staying at home (Fig 3). In Venn diagram I (staying home more than five times per month), the proportion of the admission group characterized by both monthly clinics/hospitals visits and frequent eating out was 9% (six participants), higher than the 1% (four participants) in the outpatient group (Fig 3). Similar trends were observed in Venn Diagram II (staying home more than 10 times per month) and Venn Diagram III (staying home more than 15 times per month). The admission group characterized by both monthly clinics/hospitals visits and frequent eating out comprised eight participants (II:12% and III:13%, respectively), compared with seven participants (1%) in the outpatient group.

Among participants with more than one visit to gambling establishments per month (D) in Venn diagram I, the proportion of the admission group was slightly higher for those who also ate out frequently and staying home more than five times per month to the outpatient group (admission group vs. outpatient group: two (3%) vs. six (1%)). A similar trend was observed for participants who gambled and ate out in Venn Diagrams II/III (one participant (2%) vs. four (1%), respectively).

## Discussion

In this study we sought to use mobile phone location data to explore the relationship between behavior risk factors (primarily eating-out behavior) and hospitalization. The findings suggest that the odds of hospitalization may be higher among individuals who eat out more than eight times per month (Fig 2, S3 Table). This observation supports the potential utility of mobile phone-derived geographical data for identifying behavioral patterns associated with hospitalization. This aligns with previous research indicating that frequent eating out behavior is associated with a 20% higher risk for cardiovascular events [4,8], and these findings may help generate hypotheses about the correlation of mobility-derived behaviors to hospitalization. Interestingly, our study revealed that the adjusted OR, based on the frequency of eating out per month, exhibited a reverse U-shaped association centered around the eight-times-per-month threshold. This finding suggest that individuals who eat out infrequently might practice more health-conscious dietary habits, whereas those who eat out more frequently might engage in more social activities, potentially reflecting better mental well-being and a more socially active lifestyle.

Furthermore, Venn diagrams were used descriptively to illustrate overlapping behavioral characteristics between groups. In the admission group, a larger proportion of participants exhibited a behavioral pattern of both frequent eating out and regular hospital visits (Fig 3). These cooccurrence patterns can aid interpretation of the regression findings by highlighting real-world behavioral clustering; however, they do not quantify effect size and should not be interpreted as evidence of increased risk or causality. The observations suggest that outpatient dietary behaviors may correlate with subsequent healthcare utilization and highlight the need for further studies incorporating detailed clinical and socioeconomic data. Notably, digital devices could enable more timely characterization of lifestyle behaviors between clinical visits; whether such monitoring can support effective interventions or reduce hospitalizations warrants prospective evaluation. The overlap between gambling-venue visits and frequent eating out was small and is presented here as an exploratory finding (Fig 3). The focus on gambling reflects evidence that gamblers are more likely to develop coronary heart disease (CHD) due to stress, depression, and high alcohol or nicotine intake [19,20]. Gambling may also increase the incidence of atherosclerosis and heart disease, independent of body mass index and other factors [21]. While only a modest association was observed in this study, the findings suggest that this approach may provide a useful framework for exploring relationships between hospitalization and the behavioral patterns of patients. Nevertheless, given the limited strength of the observed association, further research is needed to confirm these findings and to clarify the underlying mechanisms. In addition, the measures derived from the location data have not been formally validated and may be subject to measurement error and bias.

Numerous previous studies have used location data from mobile devices to detect and visualize the spread of infection, population density, and traffic volume [22–29]. However, most of these GPS-based studies primarily focused on trends in population-level mobility. In contrast, the novelty of the present study lies in its focus on the relationship between behavioral patterns and health outcomes, specifically examining associations between eating out, staying at home, and visiting gambling establishments. Without GPS data, similar studies would typically rely on questionnaires or interviews. Although these approaches can capture self-reported behaviors and contextual information, they are susceptible to recall bias and may not accurately reflect actual behavioral patterns over time. These considerations support the value of mobile phone location data as a complementary approach for objectively assessing behavioral patterns in retrospective observational studies.“

### Limitations

This explanatory study had several limitations. First, the geographic information was collected from patients visiting cardiology clinics and hospitals, potentially limiting the generalizability of our findings to broader patient population. Furthermore, outpatient visits and admission were defined solely from geographical information as we lacked access to individual medical information records including medication, procedures and other clinical data. This limitation may affect our results. Second, while all participants continued outpatient treatment, suggesting potentially similar baseline comorbidities and disease severity across groups, the absence of detailed diagnostic data prevents us from definitively confirming this assumption. Underlying disease severity or deteriorating health status may be important sources of unmeasured confounding, because these factors could influence both hospitalization and behavioral patterns such as eating-out behavior. Moreover, the lack of comprehensive diagnostic, severity and other medical background information hinders our ability to rule out unadjusted confounding factors. In addition, eating-out frequency in the admission group was calculated using data from the entire observation period rather than being restricted to the pre-hospitalization period. Therefore, reverse causation may have biased the observed association, as hospitalization itself or deteriorating health status could have influenced mobility and eating-out behavior. Accordingly, the findings should be interpreted as associations between habitual eating-out patterns and hospitalization during the observation period, rather than as evidence that eating-out behavior preceded or caused hospitalization. Future research integrating healthcare data is necessary to address these limitations. Third, the study population was derived from users of the Blogwatcher platform. Although the platform aggregates data from a variety of applications used across different age groups and sexes, including navigation, retail, and lifestyle apps, the study population may not fully represent the general population. Consequently, selection bias cannot be excluded. In addition, substantial missing data for age and sex (>50%) limited our ability to adjust for demographic confounding factors, which may have influenced the observed associations. In addition, the measures derived from the location data have not been formally validated and may be subject to measurement error and bias.

## Conclusion

Our study demonstrated the potential of using mobile phone-based location data for analyzing and evaluating the health-related behaviors of individuals in a real-world setting. This study suggests that associations between behavioral patterns and hospitalization, particularly eating-out behavior, may be captured using GPS-based data. Importantly, this approach provides access to behavioral information that is typically difficult, time-consuming, and resource-intensive to obtain through conventional clinical research. By leveraging passively collected mobility data, real-world lifestyle patterns can be captured at scale, offering insights beyond those available from self-reports or routine clinical records. Although the observed associations were modest, these findings highlight the potential value of mobile phone-derived behavioral data as a complementary tool for investigating relationships between lifestyle behaviors and health outcomes. Given the exploratory nature of this observational study and the limitations in demographic and clinical information, further research is needed to validate these findings and clarify the underlying mechanisms. Further work should assess the potential error in measures derived from location data and the potential bias in analyses using these data. Overall, mobile phone location data may serve as a valuable complement to traditional clinical data in population-based health research.

## Supporting information

S1 Table Definition of analysis perspectives. (DOCX)

S2 Table Behavioral orientation of cases (events/person-month). (DOCX)

S3 Table Logistic regression analysis of behavioral patterns associated with hospitalization: eating-out behavior. (DOCX)

S4 Table Logistic regression analysis of behavioral patterns associated with hospitalization: staying at home. (DOCX)

S5 Table Logistic regression analysis of behavioral patterns associated with hospitalization: visit to gambling establishments. (DOCX)

S1 Fig The area analyzed in the study (two administrative areas in the south of Tokyo, Japan). Source: https://maps.gsi.go.jp/#11/35.588364/139.867630/&base=blank&ls=blank&disp=1&vs=c1g1j0h0k0l0u0t0z0r0s0m0f1&d=m. The base map was created using geospatial data provided by the Geospatial Information Authority of Japan (GSI)*. These data are published under the Public Data License (Version 1.0), which permits reuse and redistribution and is compatible with the Creative Commons Attribution 4.0 International (CC BY 4.0) license**. (*URL:https://www.gsi.go.jp/ENGLISH/index.html/ **https://www.digital.go.jp/en/resources/open_data/public_data_license_v1.0).
(TIF)
