# Supplementary material for: An exploratory study of patient hospitalization patterns and behavioral risk factors using mobile phone location data
Source: PLOS Digit Health. 2026 Jul 23;5(7):e0001512. doi: 10.1371/journal.pdig.0001512 (PMC13395353; doi:10.1371/journal.pdig.0001512)
Supplement: S1 Table — (DOCX) [file pdig.0001512.s001.docx]

S1 Table : Definition of analysis perspectives.

| Analysis perspectives | Definition |
| --- | --- |
| Temporary visit | More than 15 minutes stay at one location |
| Admission | At least 2 days stay of one hospital |
| Assumed residence | A location with the highest geographic information data during nighttime hours (1:00 AM to 5:00 AM). |
| Employment | A location other than the assumed residence with the highest geographic information data during daylight hours |
| Eating out | Visits based on Point of Interest(POI) data to "Restaurants" |
| Staying at home | The number of days detected at least 500 m from the 125 m mesh where the presumed residence is located (counts as one day even if the log is detected more than once in one day) were counted as days out of the home. The remaining of the days were counted as days at home. |
| Number and visits to clinics/hospitals | Visits to Showa Medical University Hospital, medical institutions where specialists certified by the Japanese Society of Cardiology work, and "Cardiology/Internal Medicine" based on POI data |
| Visits to gambling establishments | Visits based on POI data to "Pachinko parlors / Bicycle racing, horse racing, boat racing, and car racing |
