# Supplementary material for: An exploratory study of patient hospitalization patterns and behavioral risk factors using mobile phone location data
Source: PLOS Digit Health. 2026 Jul 23;5(7):e0001512. doi: 10.1371/journal.pdig.0001512 (PMC13395353; doi:10.1371/journal.pdig.0001512)
Supplement: S2 Table — (DOCX) [file pdig.0001512.s002.docx]

S2 Table. Behavioral orientation of cases (events/person-month).

|  |  | Outpatient group (n=580) | Admission group  (n＝67) | p-value |
| --- | --- | --- | --- | --- |
| Eating out | | 7.1 [9.1] | 9.9 [11.7] | 0.023 |
| Visits of supermarkets and stores | | 1.8 [8.2] | 1.7 [3.1] | 0.026 |
| Staying at home | | 16.2 [9.4] | 13.7 [10.0] | 0.030 |
| Numbers of clinics/hospitals (excluding pharmacies) | | 0.5 [0.5] | 1.6 [1.3] | <0.001 |
| Visits to clinics/hospitals  (excluding pharmacies) | | 1.0 [3.0] | 4.3 [4.9] | <0.001 |
| Numbers of pharmacies | | 1.1 [1.2] | 2.0 [1.9] | <0.001 |
| Visits to pharmacies | | 1.8 [5.9] | 3.0 [3.6] | <0.001 |
| Visits of health-related government offices | | 0.1 [1.0] | 0.1 [0.2] | 0.089 |
| Visit to gambling establishments | | 0.3 [1.2] | 0.6 [2.7] | 0.332 |

Univariate analysis (Mann-Whitney U test) was performed for the outpatient group and admission group. Data were expressed as range median or mean ± standard deviation (SD).
