# Supplementary material for: An exploratory study of patient hospitalization patterns and behavioral risk factors using mobile phone location data
Source: PLOS Digit Health. 2026 Jul 23;5(7):e0001512. doi: 10.1371/journal.pdig.0001512 (PMC13395353; doi:10.1371/journal.pdig.0001512)
Supplement: S3 Table — (DOCX) [file pdig.0001512.s003.docx]

|  |  | outpatient group (n=580) | admission group  (n＝67) | Crude OR (95%CI) | Adjusted OR (95%CI)* | p-value |
| --- | --- | --- | --- | --- | --- | --- |
| Eating out (More than 1 per month): Yes/No | | 478/102 | 60/7 | 1.83 (0.12-4.11) | 1.31 (0.53-3.23) | 0.551 |
| Eating out (More than 4 per month): Yes/No | | 312/268 | 44/23 | 1.64 (0.97-2.79) | 1.29 (0.69-2.42) | 0.432 |
| Eating out (More than 8 per month): Yes/No | | 174/406 | 34/33 | 2.40 (1.44-4.01) | 1.89 (1.05-3.43) | ＜0.05 |
| Eating out (More than 12 per month): Yes/No | | 97/483 | 19/48 | 1.97 (1.11-3.50) | 1.65 (0.87-3.15) | 0.127 |
| Eating out (More than 16 per month): Yes/No | | 52/528 | 7/60 | 1.18 (0.51-2.72) | 0.84 (0.34-2.08) | 0.712 |

S3 Table. Logistic regression analysis of behavioral patterns associated with hospitalization: eating-out behavior

OR, odds ratio; CI, confidence interval.

*Employment, staying at home, monthly clinics/hospitals visit, visit to gambling establishments.
