# Supplementary material for: An exploratory study of patient hospitalization patterns and behavioral risk factors using mobile phone location data
Source: PLOS Digit Health. 2026 Jul 23;5(7):e0001512. doi: 10.1371/journal.pdig.0001512 (PMC13395353; doi:10.1371/journal.pdig.0001512)
Supplement: S4 Table — (DOCX) [file pdig.0001512.s004.docx]

|  |  | Outpatient group (n=580) | Admission group  (n＝67) | Crude OR (95%CI) | Adjusted OR(95%CI)* | p-value |
| --- | --- | --- | --- | --- | --- | --- |
| Staying at home (More than 5 per month): Yes/No | | 498/82 | 47/20 | 0.39 (0.21-0.68) | 0.62 (0.32-1.20) | 0.156 |
| Staying at home (More than 10 per month): Yes/No | | 381/199 | 36/31 | 0.61 (0.36-1.01) | 1.00 (0.55-1.84) | 0.990 |
| Staying at home (More than 15 per month): Yes/No | | 289/291 | 29/38 | 0.77 (0.46-1.28) | 1.26 (0.70-2.26) | 0.445 |
| Staying at home (More than 20 per month): Yes/No | | 218/362 | 19/48 | 0.66 (0.38-1.15) | 0.96 (0.52-1.75) | 0.881 |
| Staying at home (More than 25 per month): Yes/No | | 146/434 | 13/54 | 0.72 (0.38-1.35) | 0.94 (0.48-1.83) | 0.849 |

S4 Table. Logistic regression analysis of behavioral patterns associated with hospitalization: staying at home

OR, odds ratio; CI, confidence interval.

*Employment, eating out, monthly clinics/hospitals visit, visit to gambling establishments.
