# Supplementary material for: An exploratory study of patient hospitalization patterns and behavioral risk factors using mobile phone location data
Source: PLOS Digit Health. 2026 Jul 23;5(7):e0001512. doi: 10.1371/journal.pdig.0001512 (PMC13395353; doi:10.1371/journal.pdig.0001512)
Supplement: S5 Table — (DOCX) [file pdig.0001512.s005.docx]

|  |  | outpatient group (n=580) | admission group  (n＝67) | Crude OR (95%CI) | Adjusted OR(95%CI)* | p-value |
| --- | --- | --- | --- | --- | --- | --- |
| Visit to gambling establishments  (More than 0.1 visit per month): Yes/No | | 216/364 | 25/42 | 1.00 (0.59-1.69) | 0.84 (0.48-1.47) | 0.540 |
| Visit to gambling establishments  (More than 0.3 visit per month): Yes/No | | 80/500 | 10/57 | 1.10 (0.54-2.24) | 1.00 (0.47-2.14) | 0.999 |
| Visit to gambling establishments  (More than 0.5 visit per month): Yes/No | | 49/531 | 7/60 | 1.26 (0.55-2.92) | 1.11 (0.45-2.71) | 0.820 |
| Visit to gambling establishments  (More than 0.8 visit per month): Yes/No | | 35/545 | 5/62 | 1.26 (0.47-3.32) | 1.21 (0.43-3.35) | 0.720 |
| Visit to gambling establishments  (More than 1 visit per month): Yes/No | | 29/551 | 5/62 | 1.53 (0.57-4.10) | 1.43 (0.51-4.06) | 0.498 |

S5 Table. Logistic regression analysis of behavioral patterns associated with hospitalization: visit to gambling establishments

OR, odds ratio; CI, confidence interval.

*Employment, eating out, staying at home, monthly clinics/hospitals visit.

**FIGURE LEGENDS**

S1 Fig. The area analyzed in the study (two administrative areas in the south of Tokyo, Japan)

Source : https://maps.gsi.go.jp/#11/35.588364/139.867630/&base=blank&ls=blank&disp=1&vs=c1g1j0h0k0l0u0t0z0r0s0m0f1&d=m
The base map was created using geospatial data provided by the Geospatial Information Authority of Japan (GSI) ^＊^.

These data are published under the Public Data License (Version 1.0), which permits reuse and redistribution and is compatible with the Creative Commons Attribution 4.0 International (CC BY 4.0) license^＊＊^.

(＊URL:https://www.gsi.go.jp/ENGLISH/index.html/＊＊https://www.digital.go.jp/en/resources/open_data/public_data_license_v1.0)
