# Supplementary figures and images for: An exploratory study of patient hospitalization patterns and behavioral risk factors using mobile phone location data
Source: PLOS Digit Health. 2026 Jul 23;5(7):e0001512. doi: 10.1371/journal.pdig.0001512 (PMC13395353; doi:10.1371/journal.pdig.0001512)

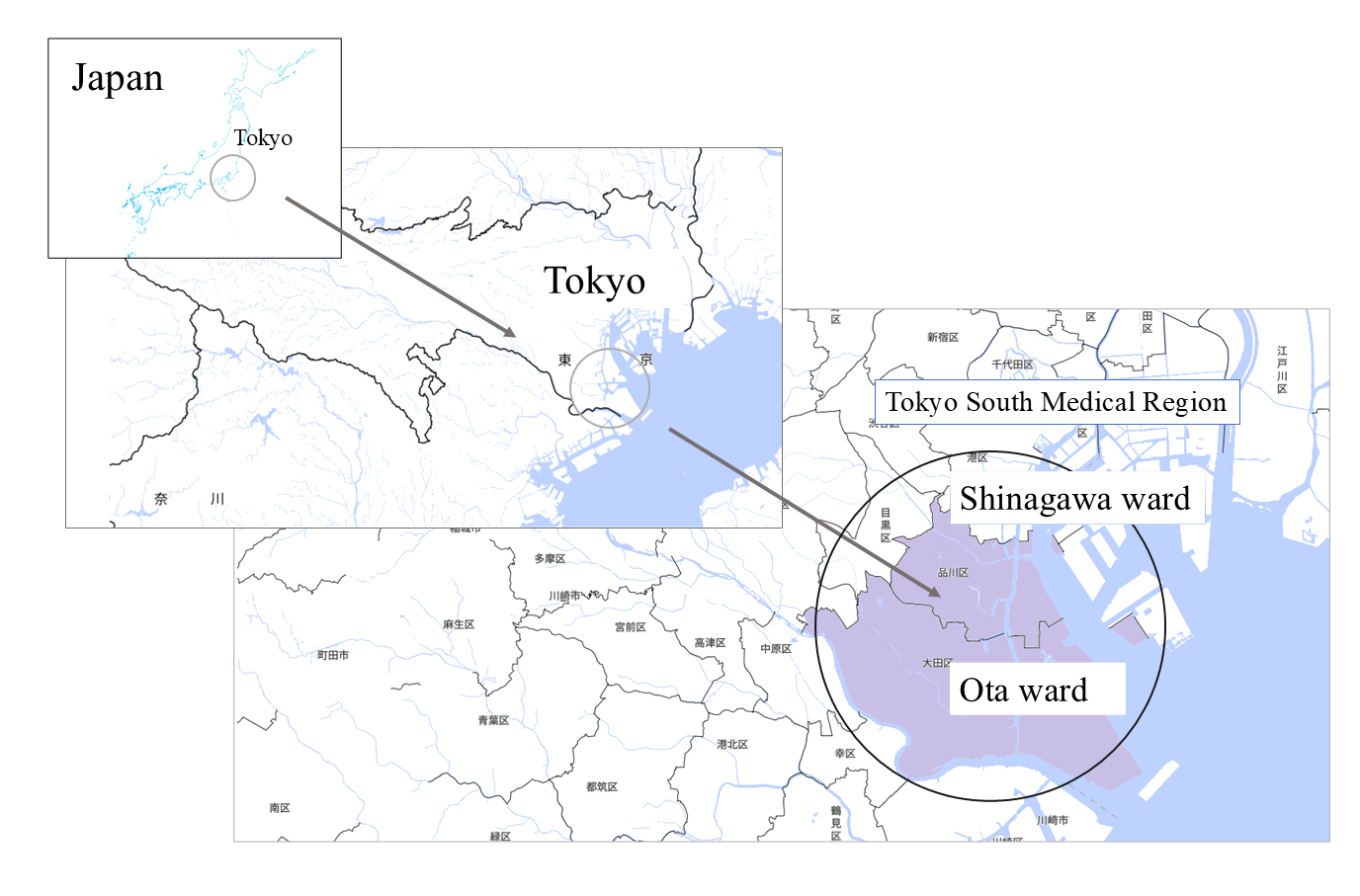

Supplement: S1 Fig — Source: https://maps.gsi.go.jp/#11/35.588364/139.867630/&base=blank&ls=blank&disp=1&vs=c1g1j0h0k0l0u0t0z0r0s0m0f1&d=m. The base map was created using geospatial data provided by the Geospatial Information Authority of Japan (GSI)*. These data are published under the Public Data License (Version 1.0), which permits reuse and redistribution and is compatible with the Creative Commons Attribution 4.0 International (CC BY 4.0) license**. (*URL:https://www.gsi.go.jp/ENGLISH/index.html/ **https://www.digital.go.jp/en/resources/open_data/public_data_license_v1.0). (TIF) [file pdig.0001512.s006.tif]
